# Barriers impeding serologic screening for celiac disease in clinically high-prevalence populations

**DOI:** 10.1186/1471-230X-14-42

**Published:** 2014-03-05

**Authors:** Erika M Barbero, Shawna L McNally, Michael C Donohue, Martin F Kagnoff

**Affiliations:** 1 Department of Medicine, Division of Gastroenterology, University of California San Diego, 9500 Gilman Drive, La Jolla, CA 92093 MC 0623D, USA; 2 Current Address: Nutrition Research Consultant, Harvard Center for Population and Development Studies, Providence, RI, USA; 3 Department of Family and Preventive Medicine, Division of Biostatistics and Bioinformatics, University of California San Diego, La Jolla, CA, USA; 4 Department of Pediatrics, Division of Gastroenterology, University of California San Diego, La Jolla, CA, USA

**Keywords:** Celiac disease, Serologic screening, Barriers to screening

## Abstract

**Background:**

Celiac disease is present in ~1% of the general population in the United States and Europe. Despite the availability of inexpensive serologic screening tests, ~85% of individuals with celiac disease remain undiagnosed and there is an average delay in diagnosis of symptomatic individuals with celiac disease that ranges from ~5.8-11 years. This delay is often attributed to the use of a case-based approach for detection rather than general population screening for celiac disease, and deficiencies at the level of health care professionals. This study aimed to assess if patient-centered barriers have a role in impeding serologic screening for celiac disease in individuals from populations that are clinically at an increased risk for celiac disease.

**Methods:**

119 adults meeting study inclusion criteria for being at a higher risk for celiac disease were recruited from the general population. Participants completed a survey/questionnaire at the William K. Warren Medical Research Center for Celiac Disease that addressed demographic information, celiac disease related symptoms (gastrointestinal and extraintestinal), family history, co-morbid diseases and conditions associated with celiac disease, and patient-centered barriers to screening for celiac disease. All participants underwent serologic screening for celiac disease using the IgA tissue transglutaminase antibody (IgA tTG) and, if positive, testing for IgA anti-endomysial antibody (IgA EMA) as a confirmatory test.

**Results:**

Two barriers to serologic testing were significant across the participant pool. These were participants not knowing they were at risk for celiac disease before learning of the study, and participants not knowing where to get tested for celiac disease. Among participants with incomes less than $25,000/year and those less than the median age, not having a doctor to order the test was a significant barrier, and this strongly correlated with not having health insurance. Symptoms and co-morbid conditions were similar among those whose IgA tTG were negative and those who tested positive.

**Conclusion:**

There are significant patient-centered barriers that impede serologic screening and contribute to the delayed detection and diagnosis of celiac disease. These barriers may be lessened by greater education of the public and health care professionals about celiac disease symptoms, risk factors, and serologic testing.

## Background

Celiac disease is characterized by small intestinal mucosal damage and nutrient malabsorption. Disease is activated in genetically susceptible individuals by dietary exposure to gluten, the term commonly used for nitrogen-rich storage proteins found in wheat, barley, and rye.

Celiac disease affects approximately 1% of the population in the U.S. (i.e. ~ 2.3 million) whereas its prevalence in the countries in Europe varies from ~0.3% in Germany to ~2.4% in Finland [1]. Celiac disease is common in parts of North Africa and areas of the Middle East [2], but it is rare in the Japanese population consistent with a marked underrepresentation of the major human leukocyte antigen (HLA) DQ alleles that are required for disease susceptibility [3-5].

Celiac disease can present with gastrointestinal tract symptoms, a wide array of extraintestinal manifestations, or both [6]. Gastrointestinal symptoms frequently include increased gas and bloating, abdominal pain, chronic diarrhea or constipation, and weight loss, whereas extraintestinal manifestations frequently include iron deficiency with or without anemia, an unexplained decrease in bone density or premature onset osteoporosis, unexplained increases in liver transaminases, depression, chronic fatigue, peripheral neuropathy, aphthous stomatitis, unexplained infertility, and dental enamel defects.

Individuals with a high risk for celiac disease include first-degree, and to a lesser extent second degree, relatives of patients with celiac disease [7,8]. In addition, individuals with co-morbid diseases such as type I diabetes mellitus, autoimmune thyroid disease, dermatitis herpetiformis, Sjogren’s disease, microscopic colitis, and autoimmune liver disease have an increased prevalence of celiac disease [8,9]. Celiac disease has also been reported to be increased in those with dental enamel hypoplasia [10], cerebellar ataxia [11], and migraine headaches [12], and in a subset of individuals with irritable bowel syndrome, although the latter has been controversial [13].

Current guidelines recommend screening for celiac disease by assaying for serum IgA antibodies to the enzyme tissue transglutaminase 2 (IgA tTG) [14,15]. The IgA anti-endomysial antibody (EMA) test often is used as a follow up confirmatory test [8]. Esophagogastroduodenoscopy with small intestinal mucosal biopsy is the current gold standard for diagnosis in adults, although recent guidelines from the European Society of Pediatric Gastroenterology, Hepatology, and Nutrition endorse the diagnosis of celiac disease in some children in the absence of small intestinal mucosal biopsy [16].

Case finding in adults at high risk for celiac disease using serologic tests was recommended by a National Institutes of Health (NIH) Consensus Conference on Celiac Disease, the American Gastroenterology Association (AGA), and by the American College of Gastroenterology (ACG) [15,17,18]. In symptomatic patients with celiac disease, early diagnosis and treatment with a strict gluten free diet alleviates most symptoms, decreases morbidity and mortality, and is associated with an improved quality of life [19-21]. It is less clear if there is an improvement in quality of life and a mortality benefit to early diagnosis and treatment of screening detected asymptomatic individuals with celiac disease [9,22,23].

Epidemiologic studies estimate that the prevalence of celiac disease may have increased by as much as four-fold since 1954 [23]. Moreover, as many as 85% of individuals with celiac disease have not been detected and diagnosed [24]. Among diagnosed celiac disease patients, the average time from symptom onset to diagnosis has ranged from 5.8 to 11 years despite the availability of sensitive and specific serologic screening tests [19,20,25]. Further, patients may carry erroneous diagnoses during those years until a correct diagnosis is made. In addition to morbidities associated with undiagnosed symptomatic patients, those with undiagnosed celiac disease were reported to have a higher mortality rate than those with negative serology for celiac disease [23,26].

Reasons for the marked under-diagnosis of celiac disease among individuals in the general population who are clinically at an increased risk for celiac disease have not been explored. Patients that have been diagnosed with celiac disease often cite perceived deficiencies in the health care system. These include, for example, a lack of physician and health care provider awareness of the wide array of symptoms and presentations of celiac disease, its association with other diseases, and current serologic screening options [27]. We hypothesized that patient-centered barriers to screening for celiac disease also play an important role in the under-detection and -diagnosis of celiac disease. We report herein on patient-centered barriers to antibody based screening for celiac disease among clinically high-risk individuals recruited from the general population.

## Methods

### Recruitment and selection of the study population

Potential study participants were recruited through flyers posted in local supermarkets, community health clinics, community centers, on the University of California, San Diego (UCSD) college campus, and on local celiac disease websites (see flyer, Additional file 1). The flyer indicated researchers at the William K. Warren Medical Research Center for Celiac Disease at UCSD were “seeking individuals who may have celiac disease to participate in a research study to investigate reasons for poor diagnosis rates among individuals who are at high risk for celiac disease”. Individuals over age 18 wanting more information and to see if they qualified for participation in the study were instructed to download an application from the web and/or to contact with the Warren Medical Research Center for Celiac Disease Clinical Studies Office via the web or telephone. Respondents were informed that the study involved providing a serum sample and completing a study questionnaire at UCSD. Those interested in participating in the study were provided a brief written application (Additional file 2) and following its submission underwent a standardized telephone screen by the clinical study coordinator to ascertain that they met study inclusion criteria or had exclusion criteria (see Additional file 3). Inclusion criteria required the applicant to have at least one qualifying risk factor for celiac disease and at least one potential barrier to diagnosis (see list of qualifying risk factors and potential barriers to diagnosis in Lists 1 and 2, respectively, below). Criteria for determining if individuals were at high risk for celiac disease were developed according to AGA guidelines [15]. Individuals were excluded if they had been screened with a “blood test” for celiac disease in the past five years, had excluded gluten from the diet within the past month, or if a 24 hour diet history indicated they were consuming minimal gluten in the diet. Individuals reporting they had been tested with a fecal, skin or sputum test for celiac disease were not excluded since such tests have not been validated for celiac disease screening. The research team made a final decision for inclusion or exclusion of each applicant after review of the written application and telephone interview information.

List 1 Qualifying risk factors

Gastrointestinal risk factors (>4 weeks and impairing quality of life)

Chronic diarrhea

Recurrent abdominal pain

Irritable bowel syndrome (diagnosed)

Bloating

Non-gastrointestinal risk factors

Autoimmune thyroid disease

Osteoporosis or bone disease diagnosed under age 50

Unexplained infertility

Sjogren’s syndrome

Dermatitis herpetiformis

Liver disease (autoimmune, primary biliary cirrhosis, or unexplained cause for abnormal liver tests)

Type 1 diabetes mellitus

Anemia not responsive to iron therapy

Other

Family history of celiac disease (1^st^ or 2^nd^ degree relative)

List 2 Qualifying potential barriers to diagnosis

I did not know where to be tested

I did not know about celiac disease before learning about the study

I did not know I was at risk for celiac disease before learning about the study

I do not have a physician

I was not been motivated to be tested until recently

My doctor does not know which test to order

I don’t have insurance

My doctor will not test me

I did not want to see a doctor to be tested

I do not want my insurance company to know the results

I did not have symptoms so I did not want to be tested

I can’t afford the cost or co-payment

I have been scared to be tested until recently

I was tested for celiac disease with a skin/fecal/saliva test and did not know I should get a blood test

I cannot take time off work to be tested

One hundred nineteen applicants met inclusion criteria (84% of telephone screened individuals) and 22 applicants (16%) were excluded according to the study exclusion criteria after the telephone interview. All 119 applicants accepted into the study kept their scheduled appointments for serologic testing and completed a self-administered study survey (Additional file 4) at the UCSD Clinical Research Center.

Members of the William K. Warren Medical Research Center at UCSD designed the study protocol. Questions for an initial pilot study questionnaire were developed by the authors with additional input from a volunteer focus group provided from the membership of the local San Diego chapter of the Celiac Support Association (CSA), the Celiac Disease Foundation (CDF), and members of the Warren Center Community Advisory Board. The latter groups suggested the addition and/or deletion of questions, and the rewording of questions based, in large measure, on what these individuals viewed from personal experience as barriers that occurred during the process of obtaining the diagnoses of celiac disease. An initial pilot study carried out by the Warren Center (S. McNally and M.F. Kagnoff, unpublished data) revealed areas for improvement in the study design, information sought in the telephone interview process, and clarity of the finally adopted questionnaire. No participants in the pilot study were included in the present study. The final questionnaire provided the option for participants to add other barriers they experienced that were not listed in the questionnaire. The UCSD Institutional Review Board approved these investigations.

### Serologic tests

Each participant donated a blood sample drawn at UCSD that was assayed for IgA tTG levels by enzyme-linked immunosorbent assay at Prometheus Laboratories, San Diego, California, and samples testing positive were further assayed for IgA EMA. Total IgA levels were not measured, per current clinical guidelines, as IgA deficiency has not been deemed common enough to warrant routine testing unless there is otherwise reason to suspect it [8]. Participants were notified of serologic test results and interpretation of the results by mail in accordance with requirements and approval of the UCSD Institutional Review Board. All subjects were encouraged to consult a physician regarding their symptoms and the test results.

### Statistical analysis

Statistical analysis of the barriers to screening was performed across the total participant group and components of the demographic subgroups using the Wilcoxon Rank Sum Test. Specifically, the five point Likert-scale responses were submitted to the Wilcoxon Rank Sum Test with one-sided alternative hypothesis median μ>3 (“agree” or “strongly agree”). Fisher’s Exact Test was used to assess the strength of the association between each barrier and demographic categories (i.e., sex, level of education, and income). *P-*values ≤ 0.05 were considered significant. Spearman’s rank correlation coefficient was used to assess the correlation between each barrier. Bias-corrected and accelerated bootstrap with 1000 replicates was used to estimate 95% confidence intervals for Spearman’s rank correlation [28].

## Results

Demographics of the study population are shown in Table 1. The mean and median age of participants was 40.9 (range 19–78) and 34.9 years respectively, with a 3:1 female to male ratio that approximates the female sex predominance in celiac disease diagnosed in adult populations [12,19,29,30]. There was no significant difference in income or education level or ethnicity between female and male participants. Annual income of participants ranged from less than $25,000 USD to greater than $100,000 USD. Approximately 2/3^rds^ of participants were employed outside the home, 8% were students and 7% were retired. A majority of participants self-identified as White/non-Hispanic (79%) or Hispanic (13.4%). The remaining participants were Asian (3), Black (3), Arab/Middle Eastern (2), or Indian (1). Thirty-three participants reported having a first-degree family member with celiac disease, 18 of which were reported to be biopsy-proven. The majority of participants learned of the study from seeing the flyer in a grocery store, on the internet, or from friends or family members who saw the flyer. Interestingly, none of the patients reported learning of the study from flyers posted at community health clinics.

**Table 1:** Summary of demographics

**Age**	
Mean	40.9
Range	19-78
Standard deviation	15.1
**Sex**	
Males	28 (23%)
Females	91 (77%)
**Income (USD)**	
<$25,000	36 (31%)
$25,000-$50,000	33 (28%)
$50,000-$100,000	31 (27%)
>$100,000	16 (14%)
**Education**	
High School/GED	11 (9%)
Some college	35 (30%)
4 year degree	37 (31%)
Graduate degree	35 (30%)
**Employment**	
Employed	77 (65%)
Unemployed^*^	41 (35%)
**Race/Ethnicity**	
Hispanic	16 (13.4%)
White non Hispanic	94 (79%)
Other	9 (7.5%)

^*^Includes unemployed able to work, unemployed not able to work, retirees, homemakers, students, and non-paid volunteers.

Participant reported gastrointestinal and extraintestinal symptoms/findings on the survey are shown in Table 2. The spectrum of symptoms closely resembled that reported by patients with documented celiac disease patients prior to diagnosis [12], and in the majority of participants the reported symptoms were present for more than six months. The most common gastrointestinal symptoms were gas and bloating, abdominal cramping or pain, diarrhea or constipation, nausea, and mouth sores. More than 1/3^rd^ of participants reported extraintestinal symptoms that included fatigue, muscle cramps or pain, joint pain, numbness or tingling in the fingers or toes (i.e. paresthesias), symptoms of depression or anxiety, recurring headaches or migraines, pruritic skin rashes, and bone pain. The most frequently cited co-morbid health problems were depression, irritable bowel syndrome, iron-deficiency anemia and autoimmune thyroid disease (Table 3). Subjectively, most participants regarded their health as good to excellent (7% excellent, 34% very good, 44% good, 12% fair, and 3% poor).

**Table 2:** Frequency of symptoms

**Symptom**	**N (%)**
^*^Gas and bloating	103 (87.3)
^*^Abdominal pain or cramping	93 (79.5)
Fatigue	93 (78.8)
^*^Diarrhea	76 (65.0)
Constipation	72 (62.1)
Muscle cramps/pain	71 (60.2)
Joint pain	68 (57.6)
Numbness or tingling in fingers or toes	62 (52.5)
Symptoms of depression	58 (49.6)
Anxiety	57 (48.3)
Recurring headaches or migraines	56 (47.9)
Nausea	50 (42.7)
Itchy skin rash	49 (41.9)
Mouth sores	40 (33.9)
Bone pain	39 (33.1)
Poor dental enamel formation	26 (22.2)
Weight loss (unintentional)	20 (17.1)
Vomiting	13 (11.1)
Infertility	12 (10.3)
Translucent-looking teeth	11 (9.5)
Seizures	3 (2.6)
Other	20 (16.8)

^*^Symptoms/signs used to qualify individuals for inclusion in the study if persisting for more than four weeks.

**Table 3:** Frequency of co-morbid health problems

**Co-morbid health problems**	**N (%)**
Depression (diagnosed)	37 (31.1)
^*^Irritable Bowel Syndrome (IBS)	32 (26.9)
Anemia caused by iron deficiency	25 (21.0)
^*^Autoimmune thyroid disease	19 (16.0)
Anemia of unknown cause	15 (12.6)
Chronic fatigue syndrome	11 (9. 2)
Fibromyalgia	11 (9.2)
^*^Bone disease, osteoporosis, or low bone density before age 50	9 (7.6)
^*^Unexplained infertility	8 (6.7)
Anemia caused by B12 or folate deficiency	7 (5.9)
^*^Sjogren’s syndrome	3 (2.5)
^*^Dermatitis herpetiformis	2 (1.7)
^*^Autoimmune hepatitis	1 (0.8)
Crohn’s disease or ulcerative colitis	1 (0.8)
Addison’s disease	0 (0)
Anemia caused by something other than B12, folate, or iron deficiency	0 (0)
Cryptogenic liver disease	0 (0)
IgA deficiency	0 (0)
Systemic lupus erythematosus (SLE)	0 (0)
^*^Primary biliary cirrhosis	0 (0)
Primary sclerosing cholangitis	0 (0)
^*^Type 1 insulin dependent diabetes	0 (0)
^*^Unexplained elevated transaminase levels	0 (0)

^*^Health problems used to qualify individuals for participation in the study.

**Figure 1:**
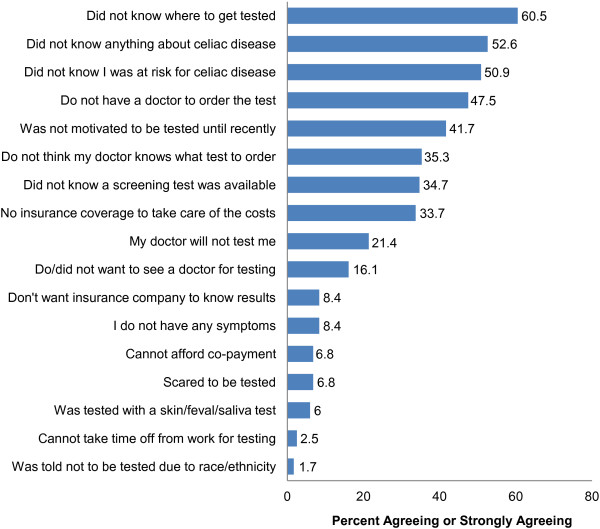
Percent of participants reporting each patient-centered barrier to diagnosis.

Two barriers were statistically significant for the population studied: “I did not know where to get tested”, and “I did not know I was at risk for celiac disease” (Table 4). Further, more than 1/3^rd^ of participants either strongly agreed or agreed with the statements, “I did not know where to get tested” (60.5%), “I did not know anything about celiac disease until recently” (52.6%), “I did not know I was at risk for celiac disease” (50.9%), “I do not have a doctor to order the test” (47.5%), “I was not motivated to get tested until recently” (41.7%), “I have a doctor, but I do not think he/she knows what test to order” (35.3%), “I knew that I may be at risk for celiac disease, but I did not know there was a screening test available” (34.7%), and “I have no insurance coverage to take care of the costs” (33.7%), whereas barriers which were cited by less than 10% of the participants were, “I do not want my health insurance company to know the results of the test”, “I do not have any symptoms and so have not wanted to get tested”, “I have insurance, but cannot afford co-payment for the test”, “I have been scared to get tested until recently or did not want to know the results”, “I was tested with a skin/fecal/saliva test and did not know that I should get the blood test”, “I cannot take time off from work to get tested at a doctor’s office”, and “I was told before that I did not need to get tested because of my race or ethnicity” (Figure 1).

**Table 4:** Frequency of barriers to screening

**Barrier statements**	**N**	**SA**^**1**^**(%)**	**A (%)**	**N (%)**	**D (%)**	**SD (%)**	**P-value**^**2**^
I did not know where to get tested.	119	23.5	37.0	17.7	9.2	12.6	<0.001
I did not know I was at risk for celiac disease.	118	21.2	29.7	17.8	17.0	14.4	0.028
I did not know anything about celiac disease until recently.	118	21.2	31.4	11.9	18.6	17.0	0.075
I do not have a doctor to order the test.	118	21.2	26.3	12.7	16.1	23.7	0.450
I was not motivated to get tested until recently.	118	16.1	25.6	9.3	16.1	22.9	0.477
I have no insurance coverage to take care of the costs.	119	21.9	11.8	8.4	22.7	35.3	0.990
I have a doctor, but I do not think he/she knows what test to order.	116	10.3	25	19.9	15.5	29.3	0.995
I knew that I may be at risk for celiac disease, but I did not know there was a screening test available.	118	11.0	23.7	17.0	20.3	28.0	0.995
I have a doctor, but he/she will not test me.	117	7.7	13.7	24.8	23.1	30.8	>0.999
I do/did not want to see a doctor for testing.	118	2.5	13.6	20.3	30.5	33.1	>0.999
I have been scared to get tested until recently or did not want to know the results.	118	1.7	5.1	17.0	36.4	39.8	>0.999
I do not have any symptoms and so have not wanted to get tested.	119	0.8	7.6	14.3	31.9	45.4	>0.999
I do not want my health insurance company to know the results of the test.	118	0.8	7.6	20.3	18.6	55.1	>0.999
I have insurance, but cannot afford co-payment for the test.	118	3.4	3.4	14.4	24.6	54.2	>0.999
I was tested with a skin/fecal/saliva test and did not know that I should get the blood test.	117	0.9	5.1	13.7	22.2	58.1	>0.999
I was told before that I did not need to get tested because of my race or ethnicity.	118	0	1.7	16.1	27.1	55.1	>0.999
I cannot take time off from work to get tested at a doctor’s office.	118	0	2.5	12.7	30.5	54.2	>0.999

^1^SA = Strongly agree; A = Agree; N = Neither; D = Disagree; SD = Strongly disagree.

^2^P values determined using Wilcoxon Rank Sum Test.

Several barriers were statistically significant for specific demographic groups (Table 5). In addition to not knowing where to get tested, those younger than the median age of 34.9 years were significantly more likely to not have a doctor than those older than the median age. Further, not knowing where to get tested, which was a significant barrier across the entire participant group, was highly significant among women (*p* ≤ 0.001, N = 91) but not in men (P = 0.314; N = 28).

**Table 5:** Significant barriers by demographic sub-population

**Sub-population**	**N**	**Significant barriers**	**P-value**
**Median age (years)**			
<34.9	60	I did not know where to get tested.	0.003
		I do not have a doctor to order the test.	0.008
>34.9	59	I did not know where to get tested.	0.012
**Sex**			
Males	28	None	>0.05
Females	91	I did not know where to get tested.	<0.001
		I did not know I was at risk for celiac disease.	0.029
**Income (USD)**			
<$25,000	34	I do not have a doctor to order the test.	0.007
		I did not know where to get tested.	0.021
$25,000-$50,000	33	I did not know where to get tested.	<0.001
$50,000-$100,000	31	I did not know I was at risk for celiac disease.	0.035
>$100,000	16	None	>0.05
**Education**			
High school/GED	11	None	>0.05
Some college	35	I did not know where to get tested.	0.031
4 year degree	37	I did not know where to get tested.	0.013
		I did not know I was at risk for celiac disease.	0.043
Graduate degree	35	I did not know I was at risk for celiac disease.	0.027
		I did not know where to get tested.	0.034
**Employment**			
Employed	77	I did not know anything about celiac disease until recently.	0.003
		I did not know where to get tested.	0.004
		I did not know I was at risk for celiac disease.	0.012
Unemployed^*^	42	I did not know where to get tested.	0.010
**Ethnicity**			
White non-Hispanic	94	I did not know where to get tested.	0.004
Hispanic	16	I did not know I was at risk for celiac disease.	0.025
Other	9	I did not know I was at risk for celiac disease.	0.015
		I did not know anything about celiac disease until recently.	0.048

^*^Includes unemployed able to work, unemployed not able to work, retirees, homemakers, students, and non-paid volunteers.

Those with annual incomes less than $25,000 differed from the higher income groups in that 65% indicated they did not have a doctor to order the test (P = 0.007), whereas the lack of a doctor to order the test was not a statistically significant barrier for the other income groups (Table 5). Consistent with that, 61.1% of individuals in the lowest income group cited the lack of health insurance as a barrier to testing (*p* = 0.06; N = 35). Not knowing where to be tested was a significant barrier for those in the lowest two income categories whereas not knowing they were at risk was a significant barrier among those in the $50,000-$100,000 annual income group. Although none of the barriers tested was statistically significant among those with annual incomes above $100,000 (Table 5), 50% of this income group cited a lack of motivation as a barrier to testing (*p* = 0.165; N = 16) making lack of motivation the most common barrier cited by this group.

Not knowing where to be tested was a significant barrier across all education levels except High School/GED, which did not have any statistically significant barriers, possibly reflecting the small sample size (Table 5). The most common barrier reported by the High School/GED demographic group was, “I did not know anything about celiac disease until recently”, with 8 out of 11 participants agreeing. A significant number of individuals with all other levels of education reported, “I did not know where to get tested” (Table 5). “I did not know I was a risk for celiac disease” was a significant barrier only for individuals with a four-year college or graduate degree.

Employed individuals and the unemployed faced an overlapping barrier in that both did not know where to get tested (Table 5), whereas only the employed significantly reported not knowing anything about celiac disease until recently and not knowing they were at risk for celiac disease.

Not knowing where to get tested was a significant barrier among White participants whereas not knowing they were at risk for celiac disease was a significant barrier among Hispanic participants. A significant number of individuals of other races/ethnicities reported not knowing they were at risk for celiac disease and not knowing about celiac disease until recently.

Strong correlations were found between several barriers suggesting validity of the questionnaire (Table 6). Subjects who reported “not knowing anything about celiac disease until recently” were also likely to cite that they did not know they were at risk for celiac disease. Individuals without insurance were also likely to cite the lack of a doctor to order the blood test, and individuals who did not know there was a screening test available also did not know where to get tested.

**Table 6:** Significant correlations between barriers

**Barrier A**	**Barrier B**	**Correlation coefficient**	**95% confidence interval**
Did not know about celiac disease	Did not know I was at risk	0.726	0.599 to 0.817
Do not have insurance	Do not have a doctor to order test	0.475	0.288 to 0.618
Did not know about screening test	Did not know where to get tested	0.364	0.175 to 0.531

Of 119 participants, three (2.5%) had a positive IgA tTG test (Table 7). All three identified themselves as White, non-Hispanic. In each case, levels were >3-fold greater than the upper limit of the laboratory’s range of normal, and each also tested positive by the confirmatory IgA EMA test. Two of three serologically positive participants reported they had at least one first-degree family member with biopsy-proven celiac disease. Their qualifying risk factors, cited barriers, symptoms, and co-morbid health problems were not distinguishable from those of the other 116 participants, who all had IgA tTG titers well below the laboratory cutoff values for normal.

**Table 7:** The risk factors, barriers, health problems, and subjective overall health of the participants with positive serologic studies

	**Qualifying risk factors on telephone interview**	**Barriers identified on survey/questionnaire**	**Symptoms on survey/questionnaire**	**Co-morbid health problems on survey/questionnaire**	**IgA tTG titer**	**Overall health**
1	Abdominal pain	Don’t know where to get tested	Abdominal pain	Autoimmune thyroid disease	29	Very good
	Autoimmune thyroid disease					
				Bone disease		
	Bone disease	Don’t think my doctor knows which test to order	Gas/bloating	Chronic fatigue syndrome		
			Nausea			
		My doctor won’t test me	Anxiety	Depression (diagnosed)		
		Don’t want to eat gluten to be tested	Symptoms of depression	Fibromyalgia		
			Fatigue			
			Bone pain			
			Skin rash			
			Muscle pain			
			Recurring headaches or migraines			
2	1st degree family member with Celiac Disease	No symptoms to cause me to want to be tested	Symptoms of depression	Anemia, unknown cause	128	Very good
		No insurance	Fatigue	Other: “chronic headaches”		
	Anemia	Not motivated to get tested	Recurring headaches or migraines			
3	1st degree family member with celiac disease	Don’t have a doctor to test me	Abdominal pain	Infertility	18	Good
			Gas/bloating	IBS		
		Don’t have insurance	Nausea	Iron deficiency anemia		
	Abdominal pain	Don’t think doctor knows which test to order	Diarrhea			
	Diarrhea		Anxiety			
	IBS		Symptoms of depression			
			Fatigue			
			Recurring headaches or Migraines			
			Muscle pain			
			Constipation			
			Poor dental enamel formation			
			Mouth sores			
			Tingling extremities			
			Joint pain			
			Weight loss (unintentional)			

## Discussion

We report there are significant patient-centered barriers that impede serologic screening and contribute to the delayed detection and diagnosis of celiac disease. The diagnosis of celiac disease is delayed from 5.8 to 11 years on average after the onset of symptoms [19,20,25]. This is the case despite readily available inexpensive serologic screening tests of high sensitivity, specificity and an increased positive predictive value when applied to populations having an increased prevalence of celiac disease [31,32].

We surveyed a group of volunteers from the general population who met study inclusion criteria for symptoms, co-morbidities and/or family history placing them clinically in populations with an increased prevalence of celiac disease. Participants had a sex ratio that paralleled that found among diagnosed celiac disease patients, symptoms and co-morbidities typical of those found in diagnosed celiac disease patients and a broad range of income and education levels [6,12,19,23].

We reasoned a priori that key patient-centered barriers would relate to a lack of knowledge about celiac disease and serologic testing. This proved to be the case as the most significant barriers among the participant group were, “I did not know I was at risk for celiac disease” and “I did not know where to get tested”. Although our study was powered to find significant barriers that affect the entire participant group, we cautiously point out several barriers that were found in some of the demographic subgroups and may indicate worthwhile avenues for further research or outreach. Regarding access to healthcare, for example, not having a doctor was a significant barrier among those with annual incomes less than $25,000 and those in the younger half of participants. Consistent with this, not having health insurance strongly correlated with participants not having a doctor. It remains to be seen to what extent recent changes in health care laws will increase the number of Americans with both insurance and a doctor, and if this will lead to a higher frequency of testing among individuals in those demographic groups.

Lack of knowledge among the public about celiac disease, its symptoms, and risk factors represented an important barrier to diagnosis. More than 50% of participants reported not knowing anything about celiac disease until recently or not knowing that they were at risk for celiac disease. As a result, at-risk patients seeing a doctor for their symptoms would be unlikely to question their physicians about celiac disease or request serologic testing. Interestingly, more than a third of participants did not think their doctor would know which test to order, suggesting low patient confidence in physician knowledge about celiac disease. Consistent with that, primary care doctors, who form the front line for detection of probable celiac disease, were in fact found to have limited knowledge of the symptoms, natural history, and methods of testing for celiac disease, implying that greater education of health care professionals about celiac disease would be beneficial [33]. To address both problems, the National Institute of Health launched a campaign in 2006 to increase celiac disease awareness among health-care professionals and the public [34]. In addition, the growing presence of gluten-free food products in grocery stores, gluten-free options on restaurant menus, and articles about celiac disease and non-celiac gluten sensitivity in the lay press also may help to increase public awareness and the patient-physician conversation on this topic.

A lack of motivation to get tested for celiac disease was self-reported in more than 40% of participants and was the most common barrier cited by those in the highest income group. Nonetheless, each participant possessed sufficient motivation to contact the Warren Medical Research Center for Celiac Disease after seeing our posted flyers, engage in a telephone screening process, and, if qualified, complete the study. Study completion required the time and the cost of travel to the UCSD campus, the time taken to complete a 20–30 minute questionnaire, and the donation of a blood sample for celiac serology. This level of motivation likely exceeds that in the general population.

Individuals with celiac disease present with a spectrum of intestinal and extraintestinal symptoms. Many of these symptoms are not debilitating, and patients often learn to “live with” their symptoms despite a decreased quality of life. This is consistent with 85% of the participants rating their overall health as good to excellent and may help explain a lack of motivation for testing. Although 10 participants claimed to not have symptoms at the time of the screening telephone interview, each of them reported symptoms consistent with celiac disease on our questionnaire.

Three of 119 (2.5%) of participants enrolled in the study, or roughly 1:40, tested positive for IgA tTG. This is consistent with a prior study in which 1 in 56 (1.7%) symptomatic individuals tested had positive serology, compared to 1:133 (0.7%) of the general population [6]. The fact that 2/33 (6%) of our participants who indicated they had a first degree relative with celiac disease tested positive for IgA tTG is consistent with the reported prevalence of celiac disease in 5-10% of first-degree relatives of celiac disease probands [7,31]. The 3 serologically positive individuals in our study would be classified as presumptive celiac disease according to AGA criteria [8], since small intestinal mucosal biopsy was not included as part of this barriers research study. We do not know whether any of the participants with symptoms and negative IgA tTG values have non-celiac gluten sensitivity [18,35,36].

AGA, ACG, and NIH recommendations favor active case-finding, rather than general population screening [8,17,18]. This strategy is more cost-effective as it increases the pretest probability and therefore the rate of positive testing, leading to fewer unnecessary procedures [9]. However, such guidelines leave the question as to whom to screen open to clinical discretion as many common symptoms and co-morbidities are associated with an increased prevalence of celiac disease. For example, a study using an active-case finding strategy to screen for celiac disease reported that 64% of survey respondents met the criteria for celiac screening [37]. This strategy will also miss many individuals with asymptomatic celiac disease and those with atypical symptoms. Notably, the case-finding strategy thus far has failed to detect and diagnose a large majority of individuals with active celiac disease. However, it is also controversial as to whether celiac disease is an appropriate disease for mass screening consistent with World Health Organization criteria [9]. Nonetheless, it is clear that new improved testing strategies for celiac disease appropriate for application in the general or select populations are required.

Several limitations of this study are worth noting. We powered the study to find significant barriers overall, and this limited the ability to definitively delineate some of the barriers within distinct demographic subgroups. Participants were not a random sample from high celiac disease prevalence populations. We designed the study such that participants were individuals from the general population who met criteria for being at a higher risk for celiac disease than those in the general population at large. Further, the study did not accurately reflect the ethnic populations residing in San Diego County as it included 13.4% of participants who self-identified as Hispanic, compared to the 33% of residents of San Diego County who reported as Hispanic by census [38]. Finally, we note that large prevalence studies have reported roughly equal numbers of men and women with celiac disease [6,23]. Nonetheless, women are more frequently diagnosed with celiac disease than men at a rate of roughly 3:1, and the population of women and men who volunteered for this study was consistent with that ratio [12,19,29,30]. This female predominance may reflect a greater likelihood for women to seek medical attention for their symptoms or press their physicians for celiac testing. In this study, our advertising strategy (e.g. in grocery stores) may have reached more women than men.

## Conclusions

The under-detection and diagnosis of celiac disease may be partially explained by patient-centered barriers related to lack of knowledge regarding celiac disease symptoms, risk factors, and screening tests. Many individuals face barriers secondary to poor access to healthcare as well. Future public awareness campaigns, improved access to healthcare, and the development of cheaper and more accurate modes of testing have the potential to increase the rate of diagnosis of this common chronic disease.

## Abbreviations

ACG: American College of Gastroenterologists; AGA: American Gastroenterological Association; EMA: Endomysial antibody; HLA: Human leukocyte antigen; IgA: Immunoglobulin A; NIH: National Institute of Health; tTG: tissue transglutaminase; UCSD: University of California, San Diego.

## Competing interests

The authors declare that they have no competing interests.

## Authors’ contributions

MFK contributed to study concept and design, data assembly, and interpretation, and drafting of the manuscript. SLM contributed to study concept and design. EMB contributed to data collection, data interpretation, and drafting of the manuscript. MCD contributed to study design and power, and data biostatistics. All authors read and approved the final manuscript.

## Authors’ information

Martin F. Kagnoff is Director of the Laboratory of Mucosal Immunology and the William K. Warren Medical Research Center for Celiac Disease at the University of California, San Diego, and an expert in mucosal immunology and the diagnosis, pathogenesis, and treatment of celiac disease. Shawna L. McNally is a Registered Dietician, Master of Public Health, Nutrition Research Consultant at the Harvard Center for Population and Development Studies, and expert in celiac disease nutrition. Michael C. Donahue is Professor of Biostatistics and Bioinformatics at the University of California, San Diego, and expert in clinical trial design and analysis. Erika M. Barbero completed these studies in fulfillment of an independent study project for the University of California, San Diego, School of Medicine.

## Pre-publication history

The pre-publication history for this paper can be accessed here:

http://www.biomedcentral.com/1471-230X/14/42/prepub

## Supplementary Material

Additional file 1 Flyer. Click here for file

Additional file 2 Application for Celiac Disease Screening Program. Click here for file

Additional file 3 Celiac Disease Telephone Screening Protocol. Click here for file

Additional file 4 Celiac Disease Screening Program Survey. Click here for file
