# Supplementary material for: Barriers impeding serologic screening for celiac disease in clinically high-prevalence populations
Source: BMC Gastroenterol. 2014 Mar 5;14:42. doi: 10.1186/1471-230X-14-42 (PMC4016507; doi:10.1186/1471-230X-14-42)
Supplement: Additional file 1 — Flyer. [file 1471-230X-14-42-S1.pdf]

Is your food making you sick?

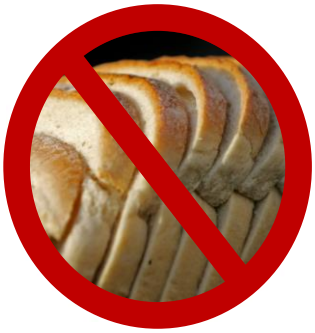

Researchers at the Wm. K. Warren Medical Research Center for Celiac Disease at the University of California, San Diego are seeking individuals who may have celiac disease to participate in a research study to investigate reasons for poor diagnosis rates among individuals who are at high risk for celiac disease.

You may be eligible to participate in this study if any of the items below apply to you.

- ✓ You have a family member diagnosed with celiac disease.
- ✓ You experience chronic diarrhea, abdominal pain, or bloating.
- ✓ You have been diagnosed with any of these health conditions.
  - Unexplained iron deficiency anemia or iron deficiency
  - Early onset (before age 50) osteoporosis or osteopenia
  - Irritable bowel syndrome
  - Unexplained infertility or miscarriage
  - Type 1 diabetes mellitus
  - Autoimmune thyroid disease
  - Autoimmune liver disease
  - Sjögren’s syndrome
  - Dermatitis herpetiformis

Must be 18 years or older to participate. For more information, and to see if you qualify for participation in this study, download an application from the center website, at http://celiaccenter.ucsd.edu, or call (858) 822-1022.

Celiac Disease Research Study
http://celiaccenter.ucsd.edu
(858) 822-1022

Celiac Disease Research Study
http://celiaccenter.ucsd.edu
(858) 822-1022

Celiac Disease Research Study
http://celiaccenter.ucsd.edu
(858) 822-1022

Celiac Disease Research Study
http://celiaccenter.ucsd.edu
(858) 822-1022

Celiac Disease Research Study
http://celiaccenter.ucsd.edu
(858) 822-1022

Celiac Disease Research Study
http://celiaccenter.ucsd.edu
(858) 822-1022

Celiac Disease Research Study
http://celiaccenter.ucsd.edu
(858) 822-1022

Celiac Disease Research Study
http://celiaccenter.ucsd.edu
(858) 822-1022

Celiac Disease Research Study
http://celiaccenter.ucsd.edu
(858) 822-1022

Celiac Disease Research Study
http://celiaccenter.ucsd.edu
(858) 822-1022
