# Supplementary material for: Barriers impeding serologic screening for celiac disease in clinically high-prevalence populations
Source: BMC Gastroenterol. 2014 Mar 5;14:42. doi: 10.1186/1471-230X-14-42 (PMC4016507; doi:10.1186/1471-230X-14-42)
Supplement: Additional file 2 — Application for Celiac Disease Screening Program. [file 1471-230X-14-42-S2.pdf]

WM. K. WARREN
MEDICAL RESEARCH CENTER
FOR CELIAC DISEASE

Application for Celiac Disease Screening Program

APPLICANT INFORMATION [please print]

Today's Date: ____________________

Name: ______________________________

Date of Birth: ____________________

Email address: ______________________________

Gender: ☐ Male ☐ Female

Home Address: ____

Telephone #(s): ______________________________ Type: ☐ Home ☐ Cell ☐ Work

______________________________ Type: ☐ Home ☐ Cell ☐ Work

______________________________ Type: ☐ Home ☐ Cell ☐ Work

How did you hear about the Warren Celiac Center screening program?

Please provide a reason for why you want to be screened for Celiac Disease.

RISK FACTORS (check all that apply to you):
☐ **I have a family member with celiac disease**.<br>*If yes, please provide relationship (i.e. father, mother, sister, brother, uncle, etc):* ____________________
☐ **I have chronic gastrointestinal complaints, such as abdominal pain, bloating/gas, diarrhea, constipation, or unexplained weight loss.**<br>*If yes, please specify most bothersome symptom(s)* ____________________
**I have non-gastrointestinal complaints associated with celiac disease such as (check all that apply):**<br>☐ Sjögren's Syndrome ☐ Autoimmune thyroid disease<br>☐ Type I Diabetes Mellitus ☐ Iron deficiency or anemia where the cause is unknown<br>☐ Unexplained Infertility/miscarriages ☐ Dermatitis Herpetiformis (a blistering, itchy skin rash)<br>☐ Osteoporosis or bone disease. If yes, please provide age at diagnosis _______<br>☐ Liver disease, either autoimmune or primary biliary cirrhosis or unexplained cause for abnormal liver tests

DIRECTIONS<br>Return completed application by JUST ONE of the following methods		
**EMAIL**<br>**Send as an e-mail attachment to**<br><br>**celiaccenter@ucsd.edu**<br>**(Please change filename to include your last name.)**	**FAX**<br>**Send to (858) 534-5691**<br><br>**(A cover sheet is not necessary.)**	**REGULAR MAIL- Send to:**<br>**UCSD - Warren Celiac Center**<br>**Attn: Screening Program**<br>**9500 Gilman Drive, MC0623D**<br>**La Jolla, CA 92093-0623**
