# Supplementary material for: Barriers impeding serologic screening for celiac disease in clinically high-prevalence populations
Source: BMC Gastroenterol. 2014 Mar 5;14:42. doi: 10.1186/1471-230X-14-42 (PMC4016507; doi:10.1186/1471-230X-14-42)
Supplement: Additional file 3 — Celiac Disease Telephone Screening Protocol. [file 1471-230X-14-42-S3.pdf]

Celiac Disease Screening Program Phone Protocol

Name: ________________________ Date of Phone Interview: ______________________

"Hi this is ______________________ calling from the Wm. K. Warren Medical Research Center for Celiac Disease. We received your application for the Celiac Disease Screening Program and are now in the process of scheduling participants for screening."

Y N Have you filled out an application?

- Yes: We will be confirming information you have already given us.
- No: We will be asking you for more information to make sure you qualify for participation.

APPLICANT INFORMATION **(collect if no application)** Date of Birth: ______________________

Email address: ____ Gender: ☐ Male ☐ Female

Home Address: ____

Telephone #(s): ____ Type: ☐ Home ☐ Cell ☐ Work

____ Type: ☐ Home ☐ Cell ☐ Work

How did you hear about the Warren Celiac Center screening program?

To be included in the study, each applicant must have one risk factor AND one barrier to screening. Once a barrier in acceptable barriers list below has been identified, skip to high risk questions.

Barriers Assessment

1. Y N Have you been screened with a blood test for celiac disease in the past?
   N=skip to question #3 Y=go to question #2
2. When were you last tested for celiac disease?
   >5 years = go to question #3 <5 years=EXCLUDE
3. Y N Before seeing our study advertisement, did you know what celiac is?
   N=Barrier identified: Go to risk factors Y=Go to next question
4. Y N Before seeing our study advertisement, did you know that you may be at risk for celiac disease?
   N=Barrier identified: Go to risk factors Y=Go to next question
5. Y N Have you had difficulty in the past getting screened for celiac disease?
   Y= Go to question #6A. N=Go to question #6B

6a. What difficulty(ies) have you had in getting screened?

6b. Why have you not been screened prior to this study?

Acceptable barriers/reasons:

- No physician
- No time to take off work
- No insurance
- Cannot afford co-payment
- Do not want health insurance company to know results
- Tested with skin/fecal/saliva test and did not know he/she should get blood test
- Doctor will not test him/her
- Doctor does not know which test to order
- Did not know about celiac disease until recently
- Did not know he/she was at risk for celiac disease until recently
- Did not think he/she was at risk due to race/ethnicity
- Did not have symptoms, so did not want to get tested
- Did not know where to get tested
- Did not want to see a doctor for testing
- Have been scared to get tested until recently
- Did not want to know the results of the test
- Not motivated to get tested until recently

Note write down all reasons provided and not listed above for discussion by research team.

Risk Factors

1. Family History (must be 1st or 2nd degree relative to qualify)

Family Member with Celiac Disease	Relationship	How were they diagnosed	When?

2. GI symptoms/signs: Lasting longer than 4 weeks duration and impairing quality of life or ability to function

Y N Chronic diarrhea
Y N Recurrent severe abdominal pain
Y N Irritable Bowel Syndrome (diagnosed)

3. Non-GI symptoms/signs:

Y N Anemia not-responsive to iron therapy
Y N Osteoporosis or bone disease (only include diagnosis under age 50)
Age at diagnosis:________.
Y N Liver disease (either autoimmune or primary biliary cirrhosis or unexplained cause for abnormal liver test)
Y N Unexplained infertility details
(continue to next page)

4. Associated conditions

Y N Type I Diabetes Mellitus
Y N Autoimmune Thyroid Disease
Y N Sjogren's syndrome
Y N Dermatitis Herpetiformis (clarify that this has been diagnoses by a health professional)
Y N Autoimmune hepatitis

Are you currently consuming gluten? Yes No

For how long have you been eating gluten? _______________________

Must be eating gluten for at least 4 weeks.

This is an absolute exclusion if "gluten free". Also need to be consuming sufficient gluten in order to participate.

In order to estimate typical gluten intake, we like to get an idea of your typical daily diet. Prompts: What did you eat in the morning? Did you have any snacks? Can you think of anything else you ate? Get amounts of gluten in food.

What did you eat during the last 24 hours:
____
____
____
____

Was this a typical day for you? If not, how did it vary from your typical day.

If applicant has a family history of celiac disease AND suffers from symptoms:
"Based on symptoms and risk factors reported during this initial application process, although you are eligible for study participation, it is also recommended that after screening you be evaluated by gastroenterologist and we will provide referral information if you like".

Explain the program

- Patient will come in to the Clinical Research Center to meet with the Warren Center nutritionist.
- The study will be explained. Informed consent will be obtained.
- Blood work will be drawn. Cost of test is $29.25. You will be mailed an invoice from Prometheus and you should pay the invoice within 30 days to obtain discounted screening test cost
- If this cost is too much, there is funding available to subsidize study participation
- Patient will be asked to fill out a survey.
- Results of the blood test will be mailed to the patient within 1-2 weeks.
- Patient will be advised to follow up with their physician. Patient may contact the Wm. K. Warren Medical Research Center for Celiac Disease at any time if they have any questions.

Schedule a date and time to come in for the program.

Date: ______________________________ **Time:** _________________

Give directions to get to CRC.
