# Supplementary material for: Barriers impeding serologic screening for celiac disease in clinically high-prevalence populations
Source: BMC Gastroenterol. 2014 Mar 5;14:42. doi: 10.1186/1471-230X-14-42 (PMC4016507; doi:10.1186/1471-230X-14-42)
Supplement: Additional file 4 — Celiac Disease Screening Program Survey. [file 1471-230X-14-42-S4.pdf]

WM. K. WARREN
MEDICAL RESEARCH CENTER
FOR CELIAC DISEASE

Celiac Disease Screening Program and Research Study

SURVEY

Thank you for agreeing to complete this survey as part of the research component of our celiac disease screening program. Filling out the survey is entirely voluntary. You are not required to answer any questions that you do not want to. The survey will remain completely anonymous. Please ask our staff research associate if you do not understand any of the questions on the survey or if you have any questions regarding this survey.

Date: ______________________ Subject ID: ______________________
FOR OFFICE USE ONLY

Background information

1) Gender: _____ Male _____ Female 2) Date of Birth: MM/DD/YY

3) Zip code or area of Southern California where you live: ________________________________

4) Number of years of formal education:

____ No high school diploma _____ Some college _____ Graduate degree

____ High school /General Educational Development (GED) diploma _____ Completed 4-yr college

5) Occupational status: Please **choose the one** which best applies to you

____ Employed Full Time (32 hr or more per week) _____ Employed Part Time (less than 32 hr per week _____ Non-paid volunteer

____ Homemaker _____ Student _____ Retired

____ Unemployed: able to work _____ Unemployed: unable to work

6) Field of employment and job title: ____

7) Annual Income: ___ <$25,000 ____ $25,000-50,000 ___ $50,000-100,000 ___ >$100,000

8) Ethnic Group: Please check your ethnic group: **you may check more than one**.

_____ American Indian/ Alaskan Native _____ Hispanic or Latino _____ Arab/ Middle Eastern

_____ Asian _____ Native Hawaiian or Other Pacific Islander _____ Indian

_____   _____   _____

_____ Black   _____ White   _____ Japanese

9) Have any of your biologic family members (family members related by blood) been diagnosed with celiac disease? If no, please skip to question #11.

_____ **Yes**   _____ **No**   _____ **Don't know / unsure**

10) Tell us about your biological family members with celiac disease. If the relationship is a twin sibling, please specify identical vs. not identical.

Person	Relationship (spouse, parent, sibling, child, aunt, uncle, first cousin)	Gender (M/F)	Age	Diagnosed with CD? (Yes/No)	Underwent biopsy? (Yes/No)	Age at diagnosis
#1						
#2						
#3						
#4						
#5						

Medical History

11) This is a checklist to identify why you have not been tested until today. Read each sentence describing barriers to testing and indicate how much you agree or disagree. *Please answer each question.*

Begin each statement with:
I have not been tested until today because…

	**Strongly Disagree**	**Disagree**	**Neither Agree nor Disagree**	**Agree**	**Strongly Agree**
I do not have a doctor to order the test.	☐	☐	☐	☐	☐
I cannot take time off from work to get tested at a doctor's office.	☐	☐	☐	☐	☐
I have no insurance coverage to take care of the costs.	☐	☐	☐	☐	☐
I have insurance, but cannot afford co-payment for the test.	☐	☐	☐	☐	☐
I do not want my health insurance company to know about the results of the test.	☐	☐	☐	☐	☐

I was tested with a skin/fecal/saliva test and did not know that I should get the blood test.	☐	☐	☐	☐	☐

(continue to next page)
(Question #11 continued from previous page)

This is a checklist to identify why you have not been screened *with a blood test* for celiac disease until today. Read each sentence describing barriers to testing and indicate how much you agree or disagree.
Please answer each question.

Begin each statement with:
I have not been tested until today because…

	**Strongly Disagree**	**Disagree**	**Neither Agree nor Disagree**	**Agree**	**Strongly Agree**
I have a doctor, but he/she will not test me.	☐	☐	☐	☐	☐
I have a doctor, but I do not think he/she knows what test to order.	☐	☐	☐	☐	☐
I did not know anything about celiac disease until recently	☐	☐	☐	☐	☐
I did not know that I was at risk for celiac disease.	☐	☐	☐	☐	☐
I knew that I may be at risk for celiac disease, but I did not know there was a screening test available.	☐	☐	☐	☐	☐
I was told before that I did not need to get tested because of my race or ethnicity.	☐	☐	☐	☐	☐
I do not have any symptoms and so have not wanted to get tested.	☐	☐	☐	☐	☐
	**Strongly Disagree**	**Disagree**	**Neither Agree nor Disagree**	**Agree**	**Strongly Agree**
I do/did not know where to get tested	☐	☐	☐	☐	☐
I do/did not want to see a doctor for testing.	☐	☐	☐	☐	☐
I have been scared to get tested until recently or did not want to know the results.	☐	☐	☐	☐	☐
	☐	☐	☐	☐	☐
I was not motivated to get tested until recently.					
	☐	☐	☐	☐	☐
Other:					

12) In the past four weeks, have you experienced the following health problems?

- For those health problems that you *have experienced in the past four weeks*, please *select the duration* of time that you have experienced the health problem.
- For those health problems that you h*ave not experienced in the past four weeks*, please select *"not applicable."*
- Please choose an answer for each health problem.

	DURATION OF HEALTH PROBLEMS THAT LED TO TESTING				
**HEALTH PROBLEM**	**Not applicable**	**2 weeks to 6 months**	**6 months to 1 year**	**1 to 3 years**	**More than 3 years**
Gastro-intestinal (GI) - related health problems:					
Abdominal pain & cramping	☐	☐	☐	☐	☐
Diarrhea	☐	☐	☐	☐	☐
Constipation	☐	☐	☐	☐	☐
Gas & bloating	☐	☐	☐	☐	☐
Nausea	☐	☐	☐	☐	☐
Vomiting	☐	☐	☐	☐	☐
Weight loss (unintentional)	☐	☐	☐	☐	☐
Health problems outside of the GI tract:					
Anxiety	☐	☐	☐	☐	☐
Bone pain	☐	☐	☐	☐	☐
Depression	☐	☐	☐	☐	☐
Fatigue	☐	☐	☐	☐	☐
Infertility	☐	☐	☐	☐	☐
Itchy skin rash	☐	☐	☐	☐	☐
Joint pain	☐	☐	☐	☐	☐
Mouth sores	☐	☐	☐	☐	☐
Muscle cramps/pain	☐	☐	☐	☐	☐
Numbness or tingling in fingers & toes	☐	☐	☐	☐	☐
Poor dental enamel formation (pitting, banding)	☐	☐	☐	☐	☐
Recurring headaches or migraines	☐	☐	☐	☐	☐
Seizures	☐	☐	☐	☐	☐
Tooth discoloration	☐	☐	☐	☐	☐
Translucent-looking teeth	☐	☐	☐	☐	☐
Other_________________	☐	☐	☐	☐	☐

13) Has ***your health care professional ever told you*** that you have the following health problems?

◻	Addison's disease
◻	Anemia caused by B12 or Folate deficiency
◻	Anemia caused by iron deficiency
◻	Anemia : I don't know the cause
◻	Anemia caused by something other than iron, B12, or folate deficiency
◻	Anxiety
◻	Autoimmune hepatitis
◻	Autoimmune thyroid disease (hyperthyroidism, hypothyroidism, Grave's disease, Hashimotos thyroiditis)
◻	Bone disease, osteoporosis or low bone mineral density test (before age of 50)
◻	Chronic Fatigue Syndrome
◻	Crohn's disease or ulcerative colitis
◻	Cryptogenic liver disease
◻	Depression
◻	Dermatitis herpetiformis
◻	Fibromyalgia
◻	IgA Deficiency
◻	Irritable Bowel Syndrome (IBS)
◻	Lupus Erythematosus (SLE)
◻	Primary biliary cirrhosis
◻	Primary sclerosing cholangitis
◻	Sjogren's syndrome
◻	Type I insulin dependent diabetes (juvenile diabetes)
◻	Unexplained elevated transaminase levels
◻	Unexplained infertility
◻	Other: ___________________________

14) How would you rate your overall health on a scale of 1-5?

5 (Excellent) _____ 4 (Very Good) _____ 3 (Good) _____ 2 (Fair) _____ 1 (Poor) _____

15) Please check any food allergies you might have:

____ I do not have any food allergies
____ Crustacean shellfish
____ Eggs
____ Fish
____ Milk
____ Peanuts
_____ Soybeans
_____ Tree nuts (almonds, walnuts, pecans)
_____ Wheat
_____ Corn
_____ Other: ___________________________

16) How did you hear about the Warren Center Screening Program for Celiac Disease?

____ Family
____ Friend
____ Internet
____ Newspaper
_____ Flyer
_____ Support group
_____ Health care provider
_____ Other: __________________________

By returning this form you have agreed to participate in this research project which involves the use of your blood test results and survey results to better understand barriers to testing in people at high risk for celiac disease. Whether you participate or not, you will receive the results of your blood test via a letter that is mailed to your home from the Wm. K. Warren Medical Research Center for Celiac Disease.

Thank you for your time and efforts!
